# Angle Measurement of Objects outside the Linear Field of View of a Strapdown Semi-Active Laser Seeker

**DOI:** 10.3390/s18061673

**Published:** 2018-05-23

**Authors:** Yongbin Zheng, Huimin Chen, Zongtan Zhou

**Affiliations:** College of Intelligence Science and Technology, National university of Defense Technology, Changsha 410073, China; fjxychm@163.com (H.C.); ztzhou@nudt.edu.cn (Z.Z.)

**Keywords:** angle measurement for all-strapdown semi-active laser seeker, four-quadrant photoelectric detector, GPS and INS, data fusion, space consistency and time consistency

## Abstract

The accurate angle measurement of objects outside the linear field of view (FOV) is a challenging task for a strapdown semi-active laser seeker and is not yet well resolved. Considering the fact that the strapdown semi-active laser seeker is equipped with GPS and an inertial navigation system (INS) on a missile, in this work, we present an angle measurement method based on the fusion of the seeker’s data and GPS and INS data for a strapdown semi-active laser seeker. When an object is in the nonlinear FOV or outside the FOV, by solving the problems of space consistency and time consistency, the pitch angle and yaw angle of the object can be calculated via the fusion of the last valid angles measured by the seeker and the corresponding GPS and INS data. The numerical simulation results demonstrate the correctness and effectiveness of the proposed method.

## 1. Introduction

Semi-active laser guidance, which has high precision and is easy to implement, is widely used in precision-guided weapons and equipment [1,2,3,4]. The core device is the semi-active laser seeker [5,6]. It receives the laser spot reflected by an object and detects the precise coordinates of the laser spot center by using a four-quadrant detector. It then calculates the pitch angle and the yaw angle between the object and the seeker’s optical axis. However, a semi-active laser seeker cannot measure the angles when the object is out of the linear field of view (FOV) [7]. This problem is even worse for a strapdown semi-active laser seeker [8]. It is still one of the bottlenecks that restricts the overall application of strapdown semi-active laser seekers.

### 1.1. Detection Principle of the Strapdown Semi-Active Laser Seeker

**Figure 1:**
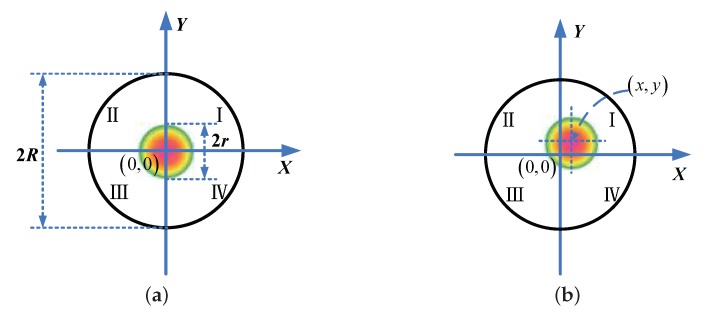
Detection principle of a four-quadrant detector. (**a**) The spot lies in the center; (**b**) The spot lies within the linear area.

The strapdown semi-active laser seeker is composed of a four-quadrant detector, optical system, circuit system and shell. The four-quadrant detector consists of four $90^{\circ }$ photosensitive sectors with the same area and the same photoelectric response [9], which are represented by *I*, II, III and IV, respectively (as shown in Figure 1). Let *R* be the radius of the photosensitive surface, *r* be the radius of the reflected laser spot, (x,y) be the coordinates of the laser spot center and $I_{i(i=I,II,III,IV)}$ be the output current of the *i*-th photosensitive sectors, which is proportional to the energy of the received laser spot. There are three cases in which (x,y) can be detected using a four-quadrant detector. The first meets the condition R<2r<2R, that is the laser spot is located in the four-quadrant detector and covers each of the four photosensitive sectors (as shown in Figure 1). Then, (x,y) can be calculated by [10,11]:

(1) $$\left\{\begin{array}{c} x=\frac{\left(I_{I}+I_{IV}\right)-\left(I_{II}+I_{III}\right)}{I_{I}+I_{II}+I_{III}+I_{IV}}\\ y=\frac{\left(I_{I}+I_{II}\right)-\left(I_{III}+I_{IV}\right)}{I_{I}+I_{II}+I_{III}+I_{IV}} \end{array}\right.$$

Obviously, (x,y)=(0,0) in the case shown in Figure 1a.

**Figure 2:**
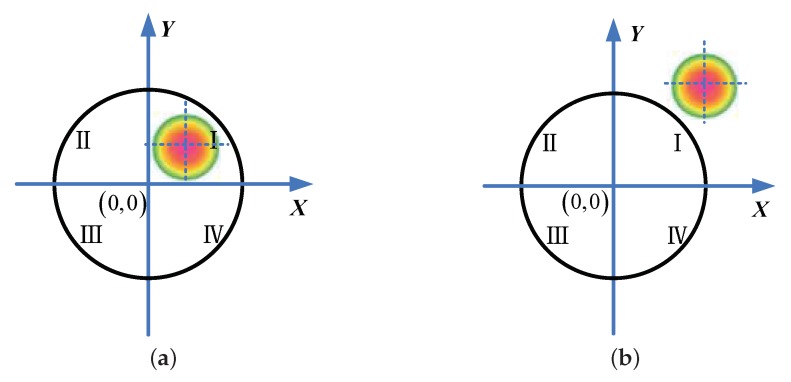
Examples of laser spot distribution on the four-quadrant detector. (**a**) The spot lies in the nonlinear area; (**b**) The spot is located outside the FOV.

The origin of the seeker’s coordinate system is the origin of the four-quadrant detector, the seeker’s X axis is along the optical axis of the optical system, and the seeker’s Y and Z axes are along the Y and X axes of the four-quadrant detector, respectively. Based on (x,y), the pitch angle ε and the yaw angle θ of the object relative to the seeker’s coordinate system can be calculated using:

(2) $$\begin{array}{c} \varepsilon =\arctan\left(\frac{y}{f}\right),\theta =\arctan\left(\frac{x}{f}\right) \end{array},$$

where *f* is the focal length of the seeker’s optical system. In this case, the satisfied area on the four-quadrant detector is called the linear area of the detector, and the corresponding FOV of the seeker is called the linear FOV. The second case is when the laser spot is within the photosensitive surface of the four-quadrant detector, but it cannot cover all four quadrants of the photosensitive sector, as shown in Figure 2a. In this case, $\left(x,y\right)$ cannot be precisely determined using Equation (1), and it can only be known in which quadrant the laser spot center is located. This area on the four-quadrant detector is called the nonlinear area, and the corresponding FOV of the seeker is called the nonlinear FOV. The third case is when the laser spot is outside the FOV of the semi-active laser seeker, as shown in Figure 2b. In this case, the four-quadrant detector cannot detect any information regarding the laser spot. Therefore, we must ensure that the object is within the linear FOV of the semi-active laser seeker.

### 1.2. Related Work

To ensure that the reflected laser spot lies in the linear area of the four-quadrant detector, the traditional semi-active laser seeker adopts a platform structure, that is the four-quadrant detector is installed on a complicated and high-precision servo control system [12]. The servo control system, which is composed of inertial measurement components and a dynamic follow-up system, can isolate the attitude movements of the seeker and ensure that the object is always located in the linear FOV of the seeker [4,13]. Although the platform-type semi-active laser seeker is a mature product, it has many disadvantages, such as a complex structure, high cost and large volume.

In recent years, the strapdown semi-active laser seeker has become one of the main development directions of the semi-active seeker [14,15]. It removes the high-precision servo control system and places the four-quadrant detector directly onto the longitudinal axis of the seeker. The advantages are its simpler structure, higher reliability, smaller size, lighter weight and lower cost [16]. The main disadvantage is that due to the detector moving with the seeker, the problem of objects going outside the linear FOV is exacerbated. The current approach is to increase the linear FOV of the seeker via a special optics design [17,18,19,20]. However, there are three shortcomings to this approach. First, the amount by which the linear area of the detector can be expanded and the seeker FOV can be increased via optics design is very limited. Second, when the linear FOV increases, the angle measurement accuracy of the seeker will decrease, which will affect the guidance accuracy of the seeker [8]. Third, increasing the FOV of the seeker results in a shortening of the detection range, and the detection range is very important for the terminal guidance of a missile. Therefore, the problem of angle measurement for a strapdown laser seeker when objects are outside the linear FOV still hinders the full application of the strapdown semi-active laser seeker.

Considering the fact that the strapdown semi-active laser seeker is equipped with GPS and inertial attitude measurement equipment on the missile [21,22], in this work, we make full use of GPS and INS data and propose an angle measurement method for the strapdown semi-active laser seeker through data fusion.

## 2. Proposed Method

When an object is outside the linear FOV, by solving the space consistency problem and the time consistency problem, the pitch angle and the yaw angle can be calculated by fusing the following data: the current GPS and inertial attitude data, the angles measured by the seeker at the last moment when the object is in the linear FOV and the corresponding GPS and inertial attitude data at that moment. The following gives the specific details of this method.

### 2.1. Definition of Variables

Assume $t_{0}$ to be the last moment at which an object is located within the linear FOV of a strapdown semi-active laser seeker, and let the pitch angle and the yaw angle of the object measured by the seeker at $t_{0}$ be $\varepsilon_{0}$ and $\theta_{0}$, respectively. Suppose that at time $t_{1}$, the object is outside the linear FOV of the seeker. Then, the pitch angle $\varepsilon_{1}$ and the yaw angle $\theta_{1}$ cannot be measured by the seeker and will be calculated using the proposed method. Our method needs the following data: the object position $O_{T}$ (longitude $\lambda_{T}$, latitude $L_{T}$ and height $h_{T}$), which is given in advance; the position of the seeker at $t_{0}$ (longitude $\lambda_{0}$, latitude $L_{0}$ and height $h_{0}$); the attitude data of the seeker with the yaw-pitch-roll rotation order at time $t_{0}$ (yaw angle $\varphi_{0}$, pitch angle $\psi_{0}$ and roll angle $\gamma_{0}$) or the quaternions at $t_{0}$($q_{0\_0},q_{1\_0},q_{2\_0},q_{3\_0}$); the position of the seeker at $t_{1}$ (longitude $\lambda_{1}$, latitude $L_{1}$ and height $h_{1}$); and theattitude data of the seeker with the yaw-pitch-roll rotation order at time $t_{1}$ (yaw angle $\varphi_{1}$, pitch angle $\psi_{1}$ and roll angle $\gamma_{1}$) or the quaternions at $t_{1}$($q_{0\_1},q_{1\_1},q_{2\_1},q_{3\_1}$). The above positions and attitude data can be obtained via GPS and INS [23,24]. The problem of time consistency can be solved by precisely aligning the above data with the corresponding time.

### 2.2. Definitions of the Coordinate Systems

To solve the space consistency problem, we define the following coordinate systems.

The Earth-centered frame $O_{e}$-$X_{e}Y_{e}Z_{e}$: The origin $O_{e}$ is the center of the Earth. The axis $O_{e}Z_{e}$ is perpendicular to the Earth’s equatorial plane and points toward the North Pole. The axis $O_{e}X_{e}$ lies in the Earth’s equatorial plane and points to the Greenwich meridian. The axis $O_{e}Y_{e}$ is perpendicular to the plane $O_{e}X_{e}Z_{e}$ and forms a right-hand coordinate system with $O_{e}X_{e}$ and $O_{e}Z_{e}$.

The local navigation frame $O_{n}$-$X_{n}Y_{n}Z_{n}$: The local navigation frame is defined as having a north-up-east order. The origin $O_{n}$ is the centroid of the seeker. The axis $O_{n}Y_{n}$ is collinear with the normal of the navigation frame’s reference ellipsoid at the penetration point. The axis $O_{n}X_{n}$ lies in the Meridian plane, is perpendicular to $O_{n}Y_{n}$ and points toward the north. The axis $O_{n}Z_{n}$ is determined according to the right-hand rule.

The body frame $O_{b}$-$X_{b}Y_{b}Z_{b}$: The origin $O_{b}$ is the centroid of the seeker. The axis $O_{b}X_{b}$ coincides with the longitudinal axis of the seeker and points toward the forward direction. The axis $O_{b}Y_{b}$ lies in the longitudinal symmetry plane of the seeker, is perpendicular to $O_{b}X_{b}$ and points upward. The axis $O_{b}Z_{b}$ is perpendicular to the $O_{b}X_{b}Y_{b}$ plane and forms a right-hand coordinate system with $O_{b}X_{b}$ and $O_{b}Y_{b}$.

The on body line-of-sight frame $O_{s}$-$X_{s}Y_{b}Z_{s}$: The origin $O_{s}$ is the centroid of the seeker. $O_{s}X_{s}$ points toward the object along the line of sight. The $O_{s}Y_{s}$ axis, which points upward, is on a plane that contains $O_{s}X_{s}$ and is perpendicular to $O_{s}X_{s}$ and the plane $O_{b}X_{b}Z_{b}$ at the same time. $O_{s}Z_{s}$ is determined by the right-hand rule.

### 2.3. Analysis and Computation of the Proposed Method

**Figure 3:**
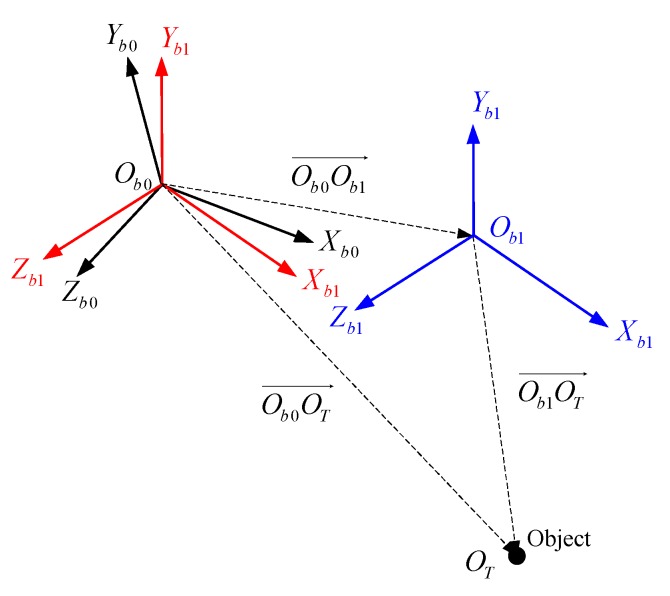
Equivalent decomposition of the seeker’s movement from $t_{0}$ to $t_{1}$.

In practice, the seeker moves with the missile all the time; thus, the position and attitude of the seeker at $t_{1}$ are different from those at $t_{0}$. As shown in Figure 3, let $O_{b0}$ be the seeker position at $t_{0}$ and $O_{b0}$-$X_{b0}Y_{b0}Z_{b0}$ be the body frame at $O_{b0}$. Moreover, let $O_{b1}$ be the seeker position at $t_{1}$ and $O_{b1}$-$X_{b1}Y_{b1}Z_{b1}$ be the body frame at $O_{b1}$. From $t_{0}$ to $t_{1}$, the body frame has both attitude movements and position movements simultaneously. Without loss of generality, we assume that the attitude movements occur first; the body frame $O_{b0}$-$X_{b0}Y_{b0}Z_{b0}$ transforms into the intermediate body frame $O_{b0}$-$X_{b1}Y_{b1}Z_{b1}$; and the, a translational movement occurs, with $O_{b0}$-$X_{b1}Y_{b1}Z_{b1}$ being translated to $O_{b1}$-$X_{b1}Y_{b1}Z_{b1}$. Therefore, the analysis and calculation required to solve the space consistency problem can be conducted in two stages.

#### 2.3.1. First Stage of the Proposed Method

In the first stage, we consider only the variation of the pitch angle and the variation of the yaw angle caused by the seeker’s attitude motion from $t_{0}$ to $t_{1}$. In this stage, the pitch angle $\varepsilon {}_{1}^{\prime }$ and yaw angle $\theta {}_{1}^{\prime }$ of the object should be calculated in the intermediate body frame $O_{b0}$-$X_{b1}Y_{b1}Z_{b1}$, with the calculation involving the following coordinate transformations: $O_{b0}$-$X_{b1}Y_{b1}Z_{b1}$ is transformed into the local navigation frame $O_{n}$-$X_{n}Y_{n}Z_{n}$; then $O_{n}$-$X_{n}Y_{n}Z_{n}$ is transformed into the body frame $O_{b0}$-$X_{b0}Y_{b0}Z_{b0}$; and finally, $O_{b0}$-$X_{b0}Y_{b0}Z_{b0}$ is transformed into the on body line-of-sight frame $O_{s}$-$X_{s}Y_{s}Z_{s}$. The specific steps are as follows.

Step 1: $O_{b0}$-$X_{b1}Y_{b1}Z_{b1}$ is transformed into $O_{n}$-$X_{n}Y_{n}Z_{n}$ by the roll-pitch-yaw rotation order with the rotations of $-\gamma_{1}$, $-\varphi_{1}$ and $-\psi_{1}$, respectively [25]. The transform matrix $C_{b1}^{n0}$ is calculated according to Equation (3).

(3) $$C_{b1}^{n0}=\left[\begin{array}{ccc} \cos\psi_{1}\cos\varphi_{1} & -\cos\gamma_{1}\sin\psi_{1}\cos\varphi_{1}+\sin\gamma_{1}\sin\varphi_{1} & \sin\gamma_{1}\sin\psi_{1}\cos\varphi_{1}+\cos\gamma_{1}\sin\varphi_{1}\\ \sin\psi_{1} & \cos\gamma_{1}\cos\varphi_{1} & -\sin\gamma_{1}\cos\varphi_{1}\\ -\cos\psi_{1}\sin\varphi_{1} & \cos\gamma_{1}\sin\psi_{1}\sin\varphi_{1}+\sin\gamma_{1}\cos\varphi_{1} & -\sin\gamma_{1}\sin\psi_{1}\sin\varphi_{1}+\cos\gamma_{1}\cos\varphi_{1} \end{array}\right]$$

To avoid the singularity problem of the Euler angles at about $90^{\circ }$, $C_{b1}^{n0}$ can be calculated based on the quaternions $q_{0\_1},q_{1\_1},q_{2\_1},q_{3\_1}$ according to Equation (4).

(4) $$C_{b1}^{n0}=\left[\begin{array}{ccc} q_{0\_1}q_{0\_1}+q_{1\_1}q_{1\_1}-q_{2\_1}q_{2\_1}-q_{3\_1}q_{3\_1} & 2\left(q_{1\_1}q_{2\_1}+q_{0\_1}q_{3\_1}\right) & 2\left(q_{1\_1}q_{3\_1}-q_{0\_1}q_{2\_1}\right)\\ 2\left(q_{1\_1}q_{2\_1}-q_{0\_1}q_{3\_1}\right) & q_{0\_1}q_{0\_1}-q_{1\_1}q_{1\_1}+q_{2\_1}q_{2\_1}-q_{3\_1}q_{3\_1} & 2\left(q_{2\_1}q_{3\_1}+q_{0\_1}q_{1\_1}\right)\\ 2\left(q_{1\_1}q_{3\_1}+q_{0\_1}q_{2\_1}\right) & 2\left(q_{2\_1}q_{3\_1}-q_{0\_1}q_{1\_1}\right) & q_{0\_1}q_{0\_1}-q_{1\_1}q_{1\_1}-q_{2\_1}q_{2\_1}+q_{3\_1}q_{3\_1} \end{array}\right]$$

Step 2: $O_{n}$-$X_{n}Y_{n}Z_{n}$ is transformed into $O_{b0}$-$X_{b0}Y_{b0}Z_{b0}$ by the yaw-pitch-roll rotation order with rotations of $\psi_{0}$, $\varphi_{0}$ and $\gamma_{0}$, respectively. The transform matrix $C_{n0}^{b0}$ is calculated according to Equation (5).

(5) $$C_{n0}^{b0}=\left[\begin{array}{ccc} \cos\psi_{0}\cos\varphi_{0} & \sin\psi_{0} & -\cos\psi_{0}\sin\varphi_{0}\\ -\cos\gamma_{0}\sin\psi_{0}\cos\varphi_{0}+\sin\gamma_{0}\sin\varphi_{0} & \cos\gamma_{0}\cos\varphi_{0} & \cos\gamma_{0}\sin\psi_{0}\sin\varphi_{0}+\sin\gamma_{0}\cos\varphi_{0}\\ \sin\gamma_{0}\sin\psi_{0}\cos\varphi_{0}+\cos\gamma_{0}\sin\varphi_{0} & -\sin\gamma_{0}\cos\varphi_{0} & -\sin\gamma_{0}\sin\psi_{0}\sin\varphi_{0}+\cos\gamma_{0}\cos\varphi_{0} \end{array}\right]$$

Similar to Step 1, $C_{n0}^{b0}$ can be calculated based on the quaternions $q_{0\_0},q_{1\_0},q_{2\_0},q_{3\_0}$ according to Equation (6):

(6) $$C_{b0}^{n0}=\left[\begin{array}{ccc} q_{0\_0}q_{0\_0}+q_{1\_0}q_{1\_0}-q_{2\_0}q_{2\_0}-q_{3\_0}q_{3\_0} & 2\left(q_{1\_0}q_{2\_0}-q_{0\_0}q_{3\_0}\right) & 2\left(q_{1\_0}q_{3\_0}+q_{0\_0}q_{2\_0}\right)\\ 2\left(q_{1\_0}q_{2\_0}+q_{0\_0}q_{3\_0}\right) & q_{0\_0}q_{0\_0}-q_{1\_0}q_{1\_0}+q_{2\_0}q_{2\_0}-q_{3\_0}q_{3\_0} & 2\left(q_{2\_0}q_{3\_0}-q_{0\_0}q_{1\_0}\right)\\ 2\left(q_{1\_0}q_{3\_0}-q_{0\_0}q_{2\_0}\right) & 2\left(q_{2\_0}q_{3\_0}+q_{0\_0}q_{1\_0}\right) & q_{0\_0}q_{0\_0}-q_{1\_0}q_{1\_0}-q_{2\_0}q_{2\_0}+q_{3\_0}q_{3\_0} \end{array}\right]$$

Step 3: $O_{b0}$-$X_{b0}Y_{b0}Z_{b0}$ is transformed into $O_{s}$-$X_{s}Y_{s}Z_{s}$ by the pitch-yaw rotation order with rotations of $\varepsilon_{0}$ and $\theta_{0}$. The transform matrix $C_{b0}^{s}$ is:

(7) $$C_{b0}^{s}=\left[\begin{array}{ccc} \cos\theta_{0}\cos\varepsilon_{0} & \cos\theta_{0}\sin\varepsilon_{0} & -\sin\theta_{0}\\ -\sin\varepsilon_{0} & \cos\varepsilon_{0} & 0\\ \sin\theta_{0}\cos\varepsilon_{0} & \sin\theta_{0}\sin\varepsilon_{0} & \cos\theta_{0} \end{array}\right]$$

To summarize, we can obtain the transformation matrix from $O_{b0}$-$X_{b1}Y_{b1}Z_{b1}$ to $O_{s}$-$X_{s}Y_{s}Z_{s}$ a:s

(8) $$C_{b0}^{s1}=C_{b0}^{s}\cdot C_{n0}^{b0}\cdot C_{b1}^{n0}$$

Step 4: According to Equation (8), we can calculate the pitch angle $\varepsilon {}_{1}^{\prime }$ using [25]:

(9) $$\varepsilon {}_{1}^{\prime }=\left\{\begin{array}{cc} \arctan\left(\frac{C_{b0}^{S1}\left[1,2\right]}{C_{b0}^{S1}\left[1,1\right]}\right), & \mathrm{if}\;C_{b0}^{S1}\left[1,2\right]\leq 0\;\mathrm{and}\;C_{b0}^{S1}\left[1,1\right]>-C_{b0}^{S1}\left[1,2\right]\\ -\arctan\left(\frac{C_{b0}^{S1}\left[1,1\right]}{C_{b0}^{S1}\left[1,2\right]}\right)+\frac{\pi }{2}, & \mathrm{if}\;C_{b0}^{S1}\left[1,1\right]>0\;\mathrm{and}\;C_{b0}^{S1}\left[1,1\right]\leq -C_{b0}^{S1}\left[1,2\right]\\ -\arctan\left(\frac{C_{b0}^{S1}\left[1,1\right]}{C_{b0}^{S1}\left[1,2\right]}\right)-\frac{\pi }{2}, & \mathrm{if}\;C_{b0}^{S1}\left[1,1\right]\leq 0\;\mathrm{and}\;C_{b0}^{S1}\left[1,1\right]>C_{b0}^{S1}\left[1,2\right]\\ \arctan\left(\frac{C_{b0}^{S1}\left[1,2\right]}{C_{b0}^{S1}\left[1,1\right]}\right)-\pi , & \mathrm{if}\;C_{b0}^{S1}\left[1,2\right]<0\;\mathrm{and}\;C_{b0}^{S1}\left[1,1\right]\leq C_{b0}^{S1}\left[1,2\right]\\ \arctan\left(\frac{C_{b0}^{S1}\left[1,2\right]}{C_{b0}^{S1}\left[1,1\right]}\right)+\pi , & \mathrm{if}\;C_{b0}^{S1}\left[1,2\right]\geq 0\;\mathrm{and}\;-C_{b0}^{S1}\left[1,1\right]>C_{b0}^{S1}\left[1,2\right]\\ -\arctan\left(\frac{C_{b0}^{S1}\left[1,1\right]}{C_{b0}^{S1}\left[1,2\right]}\right)+\frac{\pi }{2}, & \mathrm{if}\;C_{b0}^{S1}\left[1,1\right]<0\;\mathrm{and}\;-C_{b0}^{S1}\left[1,1\right]\leq C_{b0}^{S1}\left[1,2\right]\\ -\arctan\left(\frac{C_{b0}^{S1}\left[1,1\right]}{C_{b0}^{S1}\left[1,2\right]}\right)+\frac{\pi }{2}, & \mathrm{if}\;C_{b0}^{S1}\left[1,1\right]\geq 0\;\mathrm{and}\;C_{b0}^{S1}\left[1,1\right]<C_{b0}^{S1}\left[1,2\right]\\ \arctan\left(\frac{C_{b0}^{S1}\left[1,2\right]}{C_{b0}^{S1}\left[1,1\right]}\right), & \mathrm{if}\;C_{b0}^{S1}\left[1,2\right]>0\;\mathrm{and}\;C_{b0}^{S1}\left[1,1\right]\geq C_{b0}^{S1}\left[1,2\right] \end{array}\right.,$$

and the yaw angle $\theta {}_{1}^{\prime }$ using:

(10) $$\theta {}_{1}^{\prime }=-\mathrm{arc}\sin\left(C_{b0}^{s_{1}}\left[1,3\right]\right)$$

#### 2.3.2. Second Stage of the Proposed Method

In the second stage, we analyze the variation of the pitch angle and the variation of the yaw angle caused by translating $O_{b0}$-$X_{b1}Y_{b1}Z_{b1}$ to $O_{b1}$-$X_{b1}Y_{b1}Z_{b1}$. As shown in Figure 3, to calculate the vector $\vec{O_{b1}O_{T}}$, we first need to calculate the vector $\vec{O_{b0}O_{b1}}$ and the vector $\vec{O_{b0}O_{T}}$ in frame $O_{b0}$-$X_{b1}Y_{b1}Z_{b1}$. The steps of calculating $\vec{O_{b0}O_{b1}}$ in $O_{b0}$-$X_{b1}Y_{b1}Z_{b1}$ are as follows.

Step 1: Calculate the radius $R_{W0}$ of curvature in the prime vertical of the Earth and the radius $R_{N0}$ of curvature in the meridian of the Earth at $O_{b0}$ by:

(11) $$\left\{\begin{array}{cc} R_{W0} & =\frac{a_{e}^{2}}{{\left({a_{e}}^{2}\cos^{2}L_{0}+{b_{e}}^{2}\sin^{2}L_{0}\right)}^{1/2}}\\ R_{N0} & =R_{w0}\frac{b_{e}^{2}}{a_{e}^{2}} \end{array}\right.,$$

and calculate the radius $R_{W1}$ of curvature in the prime vertical of the Earth and the radius $R_{N1}$ of curvature in the meridian of the Earth at $O_{b1}$ by:

(12) $$\left\{\begin{array}{cc} R_{W1} & =\frac{a_{e}^{2}}{{\left({a_{e}}^{2}\cos^{2}L_{1}+{b_{e}}^{2}\sin^{2}L_{1}\right)}^{1/2}}\\ R_{N1} & =R_{w1}\frac{b_{e}^{2}}{a_{e}^{2}} \end{array}\right.,$$

where $a_{e}=$ 6,378,140 m and be=ae(1−11298.257)298.257) are the lengths of the Earth’s long and short half-axles, respectively.

Step 2: Calculate the coordinates ${\left[e_{x0},e_{y0},e_{z0}\right]}^{⊤}$ of $O_{b0}$ in the Earth-centered frame $O_{e}$-$X_{e}Y_{e}Z_{e}$ using:

(13) $$\left\{\begin{array}{cc} e_{x0} & =\left(R_{w0}+h_{0}\right)\cos L_{0}\cos\lambda_{0}\\ e_{y0} & =\left(R_{w0}+h_{0}\right)\cos L_{0}\sin\lambda_{0}\\ e_{z0} & =\left(R_{N0}+h_{0}\right)\sin L_{0} \end{array}\right.,$$

and the coordinates ${\left[e_{x1},e_{y1},e_{z1}\right]}^{⊤}$ of $O_{b1}$ in $O_{e}$-$X_{e}Y_{e}Z_{e}$ using:

(14) $$\left\{\begin{array}{cc} e_{x1} & =\left(R_{W1}+h_{1}\right)\cos L_{1}\cos\lambda_{1}\\ e_{y1} & =\left(R_{W1}+h_{1}\right)\cos L_{1}\sin\lambda_{1}\\ e_{z1} & =\left(R_{W1}+h_{1}\right)\sin L_{1} \end{array}\right.$$

Step 3: Calculate the transformation matrix $C_{e}^{n0}$ from the Earth-centered frame $O_{e}$-$X_{e}Y_{e}Z_{e}$ to the local navigation frame $O_{n}$-$X_{n}Y_{n}Z_{n}$ at $O_{b0}$ by:

(15) $$C_{e}^{n0}=\left[\begin{array}{ccc} -\sin L_{0}\cos\lambda_{0} & -\sin L_{0}\sin\lambda_{0} & \cos L_{0}\\ \cos L_{0}\cos\lambda_{0} & \cos L_{0}\sin\lambda_{0} & \sin L_{0}\\ -\sin\lambda_{0} & \cos\lambda_{0} & 0 \end{array}\right]$$

Step 4: Calculate the transformation matrix $C_{n0}^{b1}$ from the local navigation frame at $O_{b0}$ to $O_{b0}$-$X_{b1}Y_{b1}Z_{b1}$ by:

(16) $$C_{n0}^{b1}={\left(C_{b1}^{n0}\right)}^{⊤}$$

From Step 1 to Step 4, $\vec{O_{b0}O_{b1}}$ can be obtained by:

(17) $$\vec{O_{b0}O_{b1}}=C_{n0}^{b1}\cdot C_{e}^{n0}\cdot \left[\begin{array}{c} e_{x1}-e_{x0}\\ e_{y1}-e_{y0}\\ e_{z1}-e_{z0} \end{array}\right]$$

The vector $\vec{O_{b0}O_{T}}$ in frame $O_{b0}$-$X_{b0}Y_{b0}Z_{b0}$ is calculated as follows.

Step 1: Calculate the radius $R_{WT}$ of curvature in the prime vertical of the Earth and the radius $R_{NT}$ of curvature in the meridian of the Earth at the object position $O_{T}$ by:

(18) $$\left\{\begin{array}{cc} R_{WT} & =\frac{a_{e}^{2}}{{\left({a_{e}}^{2}\cos^{2}L_{T}+{b_{e}}^{2}\sin^{2}L_{T}\right)}^{1/2}}\\ R_{NT} & =R_{wT}\frac{b_{e}^{2}}{a_{e}^{2}} \end{array}\right.$$

Step 2: Calculate the coordinates ${\left[e_{xT},e_{yT},e_{zT}\right]}^{⊤}$ of $O_{T}$ in the Earth-centered frame by:

(19) $$\left\{\begin{array}{cc} e_{xT} & =\left(R_{wT}+h_{T}\right)\cos L_{T}\cos\lambda_{T}\\ e_{yT} & =\left(R_{wT}+h_{T}\right)\cos L_{T}\sin\lambda_{T}\\ e_{zT} & =\left(R_{NT}+h_{T}\right)\sin L_{T} \end{array}\right.$$

Step 3: Calculate the distance $Dis_{t_{0}}$ between the seeker and the object at $t_{0}$ by:

(20) $$Dis_{t_{0}}=\sqrt{{\left(e_{x0}-e_{xT}\right)}^{2}+{\left(e_{y0}-e_{yT}\right)}^{2}+{\left(e_{z0}-e_{zT}\right)}^{2}}$$

Step 4: Since the 3-2 order pitch angle and yaw angle of the object in $O_{b0}$-$X_{b1}Y_{b1}Z_{b1}$ are $\varepsilon {}_{1}^{\prime }$ and $\theta {}_{1}^{\prime }$, respectively, $\vec{O_{b0}O_{T}}$ is obtained by:

(21) $$\vec{O_{b0}O_{T}}=\left[\begin{array}{c} Dis\_t_{0}\cdot \cos\theta {}_{1}^{\prime }\cdot \cos\varepsilon {}_{1}^{\prime }\\ Dis\_t_{0}\cdot \cos\theta {}_{1}^{\prime }\cdot \sin\varepsilon {}_{1}^{\prime }\\ -Dis\_t_{0}\cdot \sin\theta {}_{1}^{\prime } \end{array}\right]$$

Step 5: The vector $\vec{O_{b1}O_{T}}$ is calculated using:

(22) $$\vec{O_{b1}O_{T}}=\vec{O_{b0}O_{T}}-\vec{O_{b0}O_{b1}}$$

Let the three components of $\vec{O_{b1}O_{T}}$ be $\Delta_{x\_t1}$, $\Delta_{y\_t1}$ and $\Delta_{z\_t1}$; then, the pitch angle $\varepsilon_{1}$ and the yaw angle $\theta_{1}$ of the object at time $t_{1}$ are finally determined as [25]:

(23) $$\varepsilon_{1}=\left\{\begin{array}{cc} \arctan\left(\frac{\Delta_{y\_t1}}{\Delta_{x\_t1}}\right), & \mathrm{if}\;\Delta_{y\_t1}\leq 0\;\mathrm{and}\;\Delta_{x\_t1}>-\Delta_{y\_t1}\\ -\arctan\left(\frac{\Delta_{x\_t1}}{\Delta_{y\_t1}}\right)-\frac{\pi }{2}, & \mathrm{if}\;\Delta_{x\_t1}>0\;\mathrm{and}\;\Delta_{x\_t1}\leq -\Delta_{y\_t1}\\ -\arctan\left(\frac{\Delta_{x\_t1}}{\Delta_{y\_t1}}\right)-\frac{\pi }{2}, & \mathrm{if}\;\Delta_{x\_t1}\leq 0\;\mathrm{and}\;\Delta_{x\_t1}>\Delta_{y\_t1}\\ \arctan\left(\frac{\Delta_{y\_t1}}{\Delta_{x\_t1}}\right)-\pi , & \mathrm{if}\;\Delta_{y\_t1}<0\;\mathrm{and}\;\Delta_{x\_t1}\leq \Delta_{y\_t1}\\ \arctan\left(\frac{\Delta_{y\_t1}}{\Delta_{x\_t1}}\right)+\pi , & \mathrm{if}\;\Delta_{y\_t1}\geq 0\;\mathrm{and}\;-\Delta_{x\_t1}>\Delta_{y\_t1}\\ -\arctan\left(\frac{\Delta_{x\_t1}}{\Delta_{y\_t1}}\right)+\frac{\pi }{2}, & \mathrm{if}\;\Delta_{x\_t1}<0\;\mathrm{and}\;-\Delta_{x\_t1}\leq \Delta_{y\_t1}\;\\ -\arctan\left(\frac{\Delta_{x\_t1}}{\Delta_{y\_t1}}\right)+\frac{\pi }{2}, & \mathrm{if}\;\Delta_{x\_t1}\geq 0\;\mathrm{and}\;\Delta_{x\_t1}<\Delta_{y\_t1}\;\\ \arctan\left(\frac{\Delta_{y\_t1}}{\Delta_{x\_t1}}\right), & \mathrm{if}\;\Delta_{y\_t1}>0\;\mathrm{and}\;\Delta_{x\_t1}\geq \Delta_{y\_t1} \end{array}\right.$$

and:

(24) $$\theta_{1}=\mathrm{arc}\tan\left(\frac{\Delta_{z\_t1}}{\sqrt{\Delta_{x\_t1}^{2}+\Delta_{y\_t1}^{2}}}\right)$$

## 3. Numerical Simulation Results

### 3.1. Numerical Simulation Setups

We carry out two simulation experiments using MATLAB to verify and evaluate the proposed method. The setups of the simulations are as follows: the measurement period of the seeker is 50 ms; the linear FOV of the seeker is $\pm 10^{\circ }$; and the FOV of the seeker is $\pm 20^{\circ }$. In the simulations, the seeker performs a sinusoidal-like motion to place the object at different positions within the seeker’s FOV. As shown in Table 1, the object is in the linear FOV in the first frame, and the pitch angle and yaw angle are 6.752∘ and $-7.187^{\circ }$, respectively. The object exits the linear FOV in the second frame (0.05 s) and stays in the nonlinear FOV from 0.05 s to 0.90 s. Then, the object leaves the FOV of the seeker at 0.95 s, re-enters the nonlinear FOV at 2.80 s and stays in the nonlinear FOV until 3.65 s. Next, it enters the linear FOV again and stays in the linear FOV until 5.05 s. Subsequently, the object again exits the linear FOV at 5.10 s and stays in the nonlinear FOV until 6.25 s. The object then stays outside the FOV between 6.30 s and 7.40 s before entering the nonlinear FOV at 7.45 s. In the simulations, we calculate the pitch angle and the yaw angle via the proposed method when the object exits the linear FOV of the seeker and evaluate the method’s performance by comparing it to the ground truth.

### 3.2. Numerical Simulation Results

**Figure 4:**
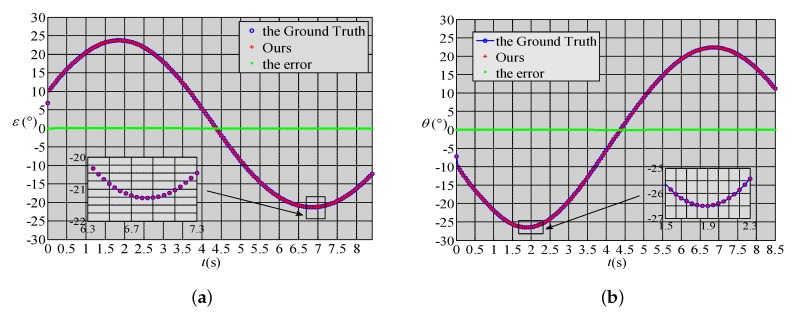
Results of the first numerical simulation. (**a**) The pitch angle results; (**b**) The yaw angle results.

The purpose of the first numerical simulation is to verify the correctness of the proposed method. In the numerical simulation, the GPS and INS data do not contain errors. The results are shown in Figure 4. Figure 4a shows the pitch angle results, while Figure 4b shows the yaw angle results. The blue `∘’ represents the ground truth, the red `+’ the result of the proposed method and the green `*’ the error between the proposed algorithm and the ground truth. It can be seen that both the pitch angle errors and the yaw angle errors are very close to zero throughout the simulation. To be more precise, the absolute values of both the pitch angle errors and the yaw angle errors are less then $1\times 10^{-6}$, which are caused by the numerical truncation of the simulation software. Therefore, this simulation result proves the correctness of the proposed method.

**Figure 5:**
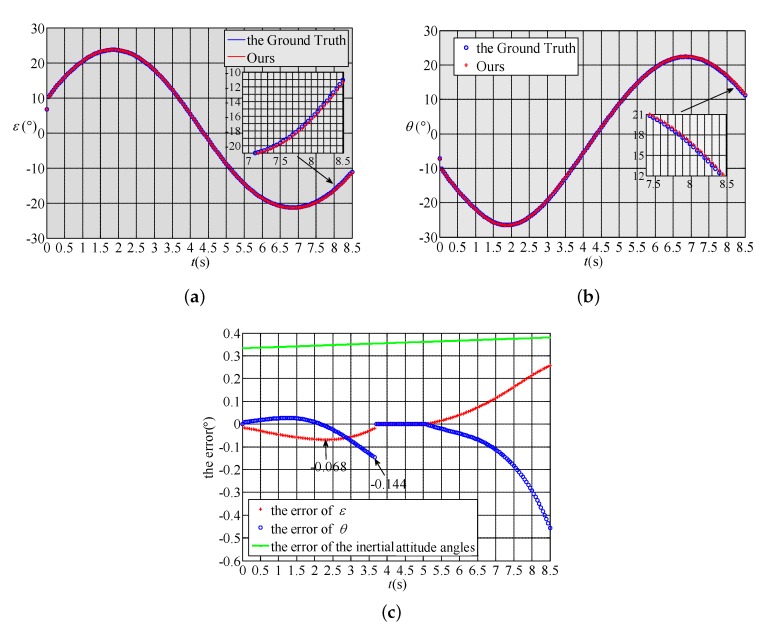
Results of the second numerical simulation. (**a**) The pitch angle results; (**b**) The yaw angle results; (**c**) The error between the proposed method and the ground truth.

The purpose of the second numerical simulation is to evaluate the angle measurement accuracy of the proposed method when the object is outside the linear FOV. In the numerical simulation, both the GPS data and INS data contain errors. The error in terms of the GPS latitude, longitude and height is 10 m. To make this numerical simulation more challenging, we assume that the INS uses a low-precision MEMS gyroscope [26,27] and that the angle drift ratio is $20^{\circ }/$h. In addition, we assume that the INS has been working for 60 s after the initial alignment. Thus, the initial attitude error of the INS is $0.333^{\circ }$. The attitude error of the INS during the numerical simulation is represented by the green curve in Figure 5c. The simulation results are shown in Figure 5: Figure 5a shows the pitch angle results; Figure 5b shows the yaw angle results; and Figure 5c shows the error between the proposed method and the ground truth. In Figure 5a,b, the blue curve represents the ground truth, while the red curve represents the result of the proposed method. In Figure 5c, the red `+’ represents the pitch angle error, and the blue `∘’ represents the yaw angle error. It can be seen that as time progresses, the object enters the nonlinear FOV or comes out of the FOV, and the angular measurement error of this method increases with the increase of the attitude error of the INS. Specifically, within the 3.6 s when the object leaves the linear FOV for the first time, as the attitude error of the INS increases to $0.354^{\circ }$, the absolute value of the pitch angle error of the proposed method increases from 0 to $0.14^{\circ }$, and the absolute value of the yaw angle error increases from 0 to $0.068^{\circ }$. Furthermore, we can conclude that under the above GPS and INS error conditions, this method can ensure that the angular measurement error is less than $0.2^{\circ }$ in the 6.5 s when the object is outside the linear FOV.

From the theoretical derivation and simulation process, it can be seen that when the object is outside the linear FOV, the angle measurement accuracy of the method increases as the GPS accuracy and INS accuracy increase and as time reduces. In practice, the accuracy of the INS is higher than $20^{\circ }/$h, and the amount of time the object spends outside the linear FOV does not exceed 3.6 s. Thus, the proposed method can achieve better angle measurement performance than exhibited during the numerical simulation.

## 4. Conclusions

To solve the problem in which a strapdown semi-active laser seeker cannot measure the angles of objects outside the linear FOV, we make full use of GPS and INS data and propose an angle measurement method based on information fusion. When an object is within the nonlinear FOV or outside the FOV, the pitch angle and the yaw angle of the object can be calculated via a fusion of the last valid angles measured by the seeker and the corresponding GPS and INS data. The numerical simulation results show that the proposed method can tolerate a certain amount of GPS and INS errors and ensure the angular measurement error is less than $0.2^{\circ }$ in the 6.5 s when the object is outside the linear FOV. In general, the proposed method is simple, accurate and effective for angle measurement of objects outside the linear FOV of a strapdown semi-active laser seeker.

## Figures and Tables

**Table 1:** Relationship between the object position and the seeker FOV.

Time *t* (s)	0	0.05–0.90	0.95–2.75	2.80–3.65	3.7–5.05	5.10–6.25	6.30–7.40	7.45–8.5
**Position relative to FOV**	Linear	Nonlinear	Outside	Nonlinear	Linear	Nonlinear	Outside	Nonlinear

## Abbreviations

The following abbreviations are used in this manuscript:

FOV	linear field of view
INS	inertial navigation system
MEMS	micro electromechanical systems

References
